# Epigenetic Mechanisms in the Neurodevelopmental Theory of Depression

**DOI:** 10.1155/2020/6357873

**Published:** 2020-04-24

**Authors:** Monika Talarowska

**Affiliations:** Department of Personality and Individual Differences, Institute of Psychology, Faculty of Educational Sciences, University of Lodz, Poland

## Abstract

The genome (genes), epigenome, and environment work together from the earliest stages of human life to produce a phenotype of human health or disease. Epigenetic modifications, including among other things: DNA methylation, modifications of histones and chromatin structure, as well as functions of noncoding RNA, are coresponsible for specific patterns of gene expression. This refers also to mental disorders, including depressive disorders. Early childhood experiences accompanied by severe stressors (considered a risk factor for depression in adult life) are linked with changes in gene expression. They include genes involved in a response to stress (hypothalamic-pituitary-adrenal axis, HPA), associated with autonomic nervous system hyperactivity and with cortical, and subcortical processes of neuroplasticity and neurodegeneration. These are, among others: gene encoding glucocorticoid receptor, FK506 binding protein 5 gene (FKBP5), gene encoding arginine vasopressin and oestrogen receptor alpha, 5-hydroxy-tryptamine transporter gene (SLC6A4), and gene encoding brain-derived neurotrophic factor. How about personality? Can the experiences unique to every human being, the history of his or her development and gene-environment interactions, through epigenetic mechanisms, shape the features of our personality? Can we pass on these features to future generations? Hence, is the risk of depression inherent in our biological nature? Can we change our destiny?

## 1. Introduction

**Figure 1:**
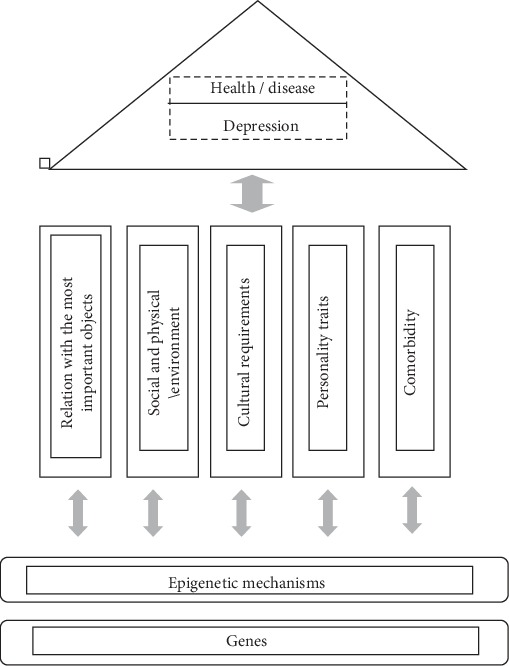
Health and disease (depression)—determinants.

The genome (genes), epigenome (chemical modifications to DNA and chromatin), and environment work together from the earliest stages of human life to create a specific phenotype of human health or disease (Figure 1). The term “epigenetics” was first used by Waddington at the end of the 1930s to describe a phenomenon related to the fact that phenotypic changes do not always go hand in hand with genotype changes [1]. Waddington suggested that the origins of development come from starting material interactions in a fertilised egg. These interactions allow something new to be created. Furthermore, he hypothesised that this is a repeating process, which leads to the formation of a new organism [1]. However, studies reporting on the importance of epigenetics in the aetiology of mental disorders have appeared only recently [2–4].

The human genome is made up of approximately 25.000 protein-coding genes [5]. Only a part of them is expressed in each cell. The aforementioned epigenetic modifications, including DNA methylation, modifications of histones and chromatin structures, as well as functions of noncoding RNA, are partially responsible for specific patterns of gene expression [6]. Each of the three processes specified above, unlike genetic changes, do not involve changes in DNA sequence [7]. These changes are affected to the largest extent by environmental factors. Through their influence on transcription of genes, they modify our phenotype [8].

Unique experiences for every human being, the history of growth, as well as interactions between genes and the environment—through epigenetic mechanisms—are considered to be a key mechanism triggering the symptoms of many somatic and mental diseases, including depression [9]. These gene–environment interactions cause epigenetic changes in gene expression patterns in which genes are switched to “on” or “off,” modifying the way cells function and thus affecting our predispositions for disease [10, 11].

Depression is a multifactorial disease. In the neurodevelopmental theory of depression [12], the authors emphasized the importance of early developmental stages for the onset of disease symptoms in adult life. This paper will focus on issues related to mother–child interactions, making an attempt to demonstrate the influence of their epigenetic mechanisms (mainly DNA methylation) leading in childhood and in adulthood to the occurrence of depressive symptoms [13].

## 2. Epigenetics in Depression

Early childhood experiences associated with severe stressors (considered a risk factor for depression in adult life) are linked with modifications in gene expression [14, 15]. Changes in the scope of gene expression affect genes involved in response to stress (hypothalamic–pituitary–adrenal axis, HPA), related to autonomic nervous system hyperactivity and cortical and subcortical processes of neuroplasticity and neurodegeneration [3], including among other genes encoding the glucocorticoid receptor, FK506-binding protein 5 (FKBP5) [16], arginine vasopressin and oestrogen receptor alpha, 5-hydroxytryptamine transporter gene (SLC6A4) [17], and brain-derived neurotrophic factors [18–20].

Story-Jovanova et al. [21] list 3 sites of methylation related to the occurrence of depression in adult life and the severity of its symptoms (7948 inhabitants of Europe participated in the study): **cg04987734** (*P* = 1.57 × 10 − 08; *n* = 11 256; CDC42BPB gene), **cg12325605** (*P* = 5.24 × 10 − 09; *n* = 11 256; ARHGEF3 gene), and intergene site CpG **cg14023999** (*P* = 5.99 × 10 − 08; *n* = 11 256; chromosome = 15q26.1). All three of these methylation sites are associated with axonal conduction. In the course of depression in elderly people (>65 years of age), a decreased level of methylation of interleukin 6 gene (IL-6), which significantly increases as a result of antidepressant treatment, may be of importance [22].

Furthermore, research teams of Park et al. [23] and Misra et al. [24] have confirmed that stress-related epigenetic changes in the following genes: NRC31, SLCA4, BDNF, FKBP5, SKA2, OXTR, LINGO3, POU3F1, ID3, TPPP, GRIN1, and ITGB1 correlate with the occurrence of depression (respectively, Misra et al., [24]). Additionally, it has been noted that negative experiences from childhood, through epigenetic changes, may have a significant impact on the effectiveness of depression pharmacotherapy [25].

### 2.1. DNA Methylation

Methylation involves a postreplication enzymatic modification of DNA (binding of the methyl group to carbon in position 5 of the cytosine ring). Cytosine methylation to 5-methylcytosine is a postreplication DNA modification that plays an important role in transcriptional silencing. This process involves a covalent bond of the methyl group with cytosine within CpG dinucleotides [26]. The DNA methylation reaction is catalysed by DNA methyltransferases, which transfer the methyl group from S-adenosyl-L-methionine to the remaining cytosine in DNA. The DNA methylation pattern is established in the early stages of embryonic growth and maintained during individual life by DNA methyltransferases (DNMTs) [4]. Under normal conditions, DNA methylation is used in the cell for silencing numerous repeated sequences, parental imprinting, and switching off the second X chromosome in female cells. Methylation of cytosine residues in DNA plays an important role in controlling the processes that determine the level of gene expression in cells during embryogenesis in mammals and later on during cell differentiation [27]. Brain function and development during all stages of life require DNA methylation in the brain [28]. It should be emphasized that the function of DNA methylation varies according to the genomic context. DNA methylation of regulatory regions that include promoters is typically linked to silencing of downstream gene expression although this effect is not absolute. In contrast, DNA methylation of gene bodies is associated with active transcription of genes [29].

### 2.2. Expression of miRNA (MicroRNA)

miRNAs are small (consisting of about 22 nucleotides), single-stranded, noncoding RNAs that act as epigenetic regulators through their effect on the posttranscriptional expression of genes [30]. They are present in plant, animal, and human cells [31]. More than 2000 mature miRNAs transcribed in the human genome have been identified so far [32]. A single miRNA can modulate thousands of genes by recognizing complementary sequences at the end of 3′UTR of the target mRNA. Endogenous miRNAs influence numerous processes taking place in cells, e.g., proliferation, DNA repair, cell differentiation, metabolism and apoptosis, as well as regulate inflammatory processes [33]. In our earlier paper, we pointed to the probable significance of miRNA-370, miRNA-411, miRNA-433, miRNA-487b, and miRNA-539 for the aetiology and course of depression [34]. Meanwhile, Narahari et al. [33] identify miRNA-16 as a posttranscriptional repressor of the serotonin transporter (SERT).

There are not many studies on the relationship between miRNAs and prenatal stressors; however, some papers indicate the significant importance of this phenomenon for the occurrence of depressive symptoms in adult life [35].

### 2.3. Modification of Histone Proteins

Posttranslational modification of histones is another epigenetic mechanism involved in the normal development and maintenance of gene expression patterns that may be important in depression aetiology [36]. Histone modifications affect the organization of the chromatin structure locally and globally, while the effects of histone modifications may affect each other antagonistically or synergistically by regulating the access of chromatin-binding proteins, which determines the transition between transcriptionally active and inactive chromatin [37]. They may lead to the activation or suppression of gene expression depending on the type of functional group connected and the type of amino acid residue which is modified [38, 39].

## 3. Depression: Can we Cheat our Destiny?

**Figure 2:**
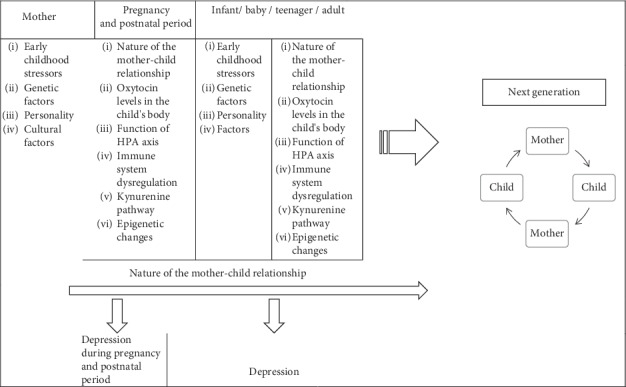
Intergenerational factors affecting the development of depression. HPA: hypothalamic–pituitary–adrenal axis.

According to Bowlby's attachment theory, the emotional bond between the mother and the child is shaped in the first year of life [40]. However, according to the assumptions of the neurodevelopmental theory of depression, this process begins much earlier because emotional experiences from three periods of life (prenatal period, early childhood, and adolescence) are of key importance for the emergence of the disease [12]. The emotional bond between mother and child not only becomes a precursor of later social relations, but it turns out that its nature influences the formation of permanent biological pathways and neurohormonal reactions, which in adult life become a “fuel” for the development of depressive and anxiety disorders, or on the contrary—they are a specific defensive mechanism in the fight against their occurrence [12] (Figure 2). Hence, if the patterns of our emotional reactions (including susceptibility to depressive disorders), through biological mechanisms, are shaped already at the stage of foetal development, are we destined for depression? Can we pass on the tendency to depressive reactions to future generations? The results of the following studies show that this is the case.

Depression during pregnancy and postpartum depression of the mother have a multidirectional effect on the occurrence of depressive disorders in the child. Not only biological factors and the related disturbance in the mother–child relationship and the bond between them (depressive mothers are less sensitive to the developmental needs of their children), but also disorders in the functioning of the family system and, finally, disorders in the marital dyad are important [41].  Table 1 shows other possible relationships between a mother's depression and the onset of depression in the child [41].

Studies conducted in the animal model (rodents studies) revealed that separation from the mother at an early stage of the offspring's life leads to permanent neuroendocrine changes, manifested in adult life in the form of cognitive, emotional, and social deficits [11, 42]. These deficits form a set of symptoms corresponding to anxiety and depressive disorders. Such behaviours are related to changes in the function of the HPA axis, both in the mother and the newborn [42]. In the human studies by Koutra et al. [43], it was demonstrated that the severity of postnatal depression symptoms in the mother and the degree of anxiety she experienced as a permanent trait of her personality were related to the quality of neuropsychological development in children. What is more, emotional closeness between parents and children during early childhood was a factor significantly affecting the volume of the cortex in the offspring's frontal gyrus area and correlated with personality traits conducive to depression in children [44].

In the meta-analysis conducted by Elwood et al. [45], a positive correlation was found between the occurrence of postnatal depression symptoms in the mother and polymorphisms of the HMNC1, COMT, MAOT, PRKCB, ESR1, and SLC6A4 genes and the presence of life events considered stressful. In those cases, when the postnatal period fell in autumn and winter months, analogous dependence concerned the BDNF gene polymorphism, and the mother's experience of violence in childhood correlated with the OXT and OXTR gene polymorphisms. According to the authors [45], women susceptible to episodes of postnatal depression are “epigenetically” more sensitive to physiological factors associated with childbirth. On the other hand, Lambert and Gressier [46] emphasize that an increased level of CRP just before and directly after the end of active labour is a risk factor of postnatal depression. In their opinion, epigenetic mechanisms may lead to pathological activity of the HPA axis and proinflammatory state.

### 3.1. Epigenetic Age

Prenatal maternal depression may induce epigenetic modifications in the DNA of the newborn child [47]. Suarez et al. [48] and Wolf et al. [49] use the term “epigenetic gestational age” (GA), which is new to psychiatry. It should be understood as the epigenetic age at birth, estimated on the basis of the level of methylation in the umbilical cord blood of the foetus. This age is currently calculated on the basis of two methods, i.e., predictor by Hannum et al. [50] (assessment of 71 CpG sites in whole blood for people aged 19-101 years) and the so-called Horvath's clock [51] (assessment of 353 CpG sites in organ tissues for people aged 0-100 years). Both of these molecular ageing biomarkers are strongly correlated with the individual's chronological age (*R* > 0.91). Chen et al. [8] say that the inclusion of epigenetic age estimates improved the ability to predict mortality [8]. In a study conducted by Suarez et al. [48], both the symptoms of depression during pregnancy and postnatal depression of mothers were correlated with a lower epigenetic age of the offspring at birth, which in turn was associated with a higher probability of mental disorders during childhood among boys. Meanwhile, Wolf et al. [49] evaluated a group of 179 war veterans from Iraq and Afghanistan. The authors emphasized that the Horvath's clock positively correlated with the severity of PTSD symptoms such as avoidance and emotional numbness. Han et al. [27] underline that in the group of people suffering from depression, there is a significantly higher epigenetic age than in the comparative group of people without the history of psychiatric treatment. Moreover, a higher epigenetic age was statistically related to the experiences of early childhood trauma.

FKBP5 is one of the proteins known as immunophilins, i.e., proteins influencing the process of immune response of the cell. In people with depression experiencing childhood violence, lower methylation was found for the gene encoding the FKBP5 protein, combined with a reduction of grey matter in the frontal gyrus region (on both sides) [16].

In a study conducted by Hein et al. [52] (19 women aged 17-29.5 years with symptoms of depression and their mothers aged 36-51 years), the level of DNA methylation in peripheral T lymphocytes in the examined mothers was associated with the severity of depression in their daughters. DNA hypermethylation in the group of mothers was also correlated with “the negative parenting”. According to the authors, this negative parenting can be a modulator between the mother's epigenome and depression of the offspring. “The negative parenting” included maternal behaviour such as hostility and aggression towards the child, indifference, neglect, and active rejection of the child (the *Parental Acceptance-Rejection Questionnaire*, PARQ). A similar effect on epigenetic changes in the offspring may also be caused by depressive symptoms in the father present after the birth of the child, often associated with the father's negative parental influences towards the offspring [53, 54] (according to Narayanan and Nærde [55], depressive symptoms in the father are more often the cause of aggressive behaviour in children). However, according to Tissot et al. [56], the symptoms of depression in the mother are of key importance for the caring behaviour of both parents.

Furthermore, the behaviour of mothers in the early postnatal period with a high level of involvement in childcare is passed on to the next generations [57]. Analogous relationships exist in the case of a mother rejecting her offspring. The fact that such behaviours occur in the adopted (and not only biological) offspring indicates a significant share of environmental factors in the acquisition of these behaviours [58]. In studies based on animal models, it is pointed out that the abovementioned behaviours are associated with changes in the methylation of genes encoding the glucocorticoid receptor observed in the hippocampal region of the examined animals [20].

In a study conducted by Stonawski et al. [19] (167 children aged 6-9 years), the diagnosis of depression during pregnancy was associated with reduced methylation in the gene encoding the glucocorticoid receptor (NR3C1), the gene encoding the mineralocorticoid receptor (NR3C2), and in the gene for the serotonin receptor (SLC6A4). In this study, genes related to the operation of the HPA axis were selected.

Lyons et al. [59] assessed the relationship between the quality of early care and markers of activation of the sympathetic nervous system and chronic inflammation (C-reactive protein level, CRP) in a sample of 52 mothers and their preschool children. The mothers who showed more attention, warmth, and support for their children's autonomy were characterized by a lower resting potential of the sympathetic nervous system and significantly lower rates of chronic inflammatory process as indicated by CRP level. CRP values were also related to a lower resting potential of the sympathetic nervous system in their children, both during relaxation and during contact with the mother or a woman unfamiliar to the child. It can therefore be concluded that the mother's supportive behaviour was conducive to the child's positive perception of new social interactions and went hand in hand with reduced excitability of the sympathetic nervous system.

Moog et al. [60] assessed the relationship between the experience of violence by mothers during childhood and the total volume of grey matter in their children's brains. It was found that the volume of grey matter in the children of mothers who were victims of violence was significantly lower than in the control group (F1.70 = 9.10; *P* = 0.004). This effect was not due to the presence of other variables that could potentially be relevant, such as the social and economic status of the mother, perinatal complications, obesity of the mother, being a victim of violence during pregnancy, severe perinatal stress, sex of the newborn child, age of the mother, or age of the child on the day of imaging examination. According to the authors, this effect may be a result of epigenetic factors.

Interesting research findings are presented by Serpeloni et al. [61], involving women who experienced violence from their intimate partners during pregnancy and their children. The level of methylation of the NR3C1 gene encoding the glucocorticoid receptor and the FKPB5 gene responsible for the organism's ability to react to stress was evaluated by regulating stress hormone expression. It was shown that being a victim of violence during pregnancy not only increases the risk of depression and anxiety symptoms in women after childbirth but is also associated with changes in the methylation level of NR3C1 and FKBP5. Similar dependencies were observed in children whose mothers experienced violence from their partners only after birth and not during pregnancy. This relationship suggests the action of epigenetic mechanisms allowing the adaptation of newborn children to unfavourable environmental conditions. It should also be noted that what is referred to as “early life adversity” (ELA), involving various forms of child abuse, such as physical violence, sexual abuse, mental and emotional abuse, and neglect, is also treated as a risk factor for depression [2].

It also turns out that prenatal exposure to SSRIs (selective serotonin reuptake inhibitors), through epigenetic mechanisms, may change the activity of the hypothalamic–pituitary–adrenal axis (HPA) [62, 63], which is particularly active in response to stress stimuli (this effect is direct and indirect, through modulation of glucocorticosteroids secretion) [64]. Serotonin has several important functions during embryonic and foetal brain development, including neuronal maturation, migration, synaptogenesis, and differentiation of neural crest cells [65], while serotonin transporter (5-HTT) was isolated in the human placenta [66]. Therefore, SSRIs crossing the blood–brain barrier of the foetus, through modification of serotonin signalling, potentially alter behaviour in childhood, adolescence, and adulthood [64]. Moreover, according to Ornoy and Koren [62], the use of SSRIs by mothers in the last months of pregnancy may cause the so-called poor neonatal adaptation syndrome, which includes irritability, excessive weeping, weak muscle tension, and respiratory disorders. However, reports on this issue are contradictory [67]. Interestingly enough, Bleker et al. [68] emphasize the positive influence of cognitive behavioural psychotherapy in women during pregnancy on the methylation level of selected genes in their children.

### 3.2. Oxytocin: Social Neuropeptide?

Oxytocin is a neurohormone—a 9-amino acid neuropeptide produced by the hypothalamus, whose activity is closely related to the previously mentioned arginine vasopressin [69, 70]. The presence of oxytocin and its receptors (OTR) is observed in the brain structures important for the establishment and maintenance of social relations and the development of depressive disorders, such as the amygdala and the hippocampus, the nucleus accumbens, and dorsal nucleus of vagus nerve [71, 72]. The target area of its operation is also the dorsal part of the cingulate gyrus and the orbitofrontal cortex [73].

Oxytocin determines the formation of attachment between mother and child [74]. Its level in the mother in the postnatal period influences the severity of separation anxiety, symptoms of depression, the image of oneself as a mother, and also shapes the anxiety-based style of attachment in the mother–child relationship [74, 75].

Krause et al. [76] emphasize that early childhood trauma in the form of violence has a negative effect on the neurohormonal oxytocin system, which manifests itself in reduced expression of the oxytocin receptor (OXTR) gene in adulthood. In children at the centre of war and military actions, a decreased level of oxytocin was associated with an increase in the immunodeficiency factor (concentration of immunoglobulin A in saliva (s-IgA) was evaluated). These variables were associated with a higher intensity of anxiety and more frequent occurrence of posttraumatic stress disorders in this group of children [77].

It is also worth noting that recent studies based on animal models indicate positive effects of antidepressants which eliminate negative effects of epigenetic mechanisms [78, 79]. However, as we mentioned earlier, the results of the research conducted so far are not clear.

### 3.3. Further Directions of Research

It should be noted that depression is the main cause of disability worldwide and more than half of patients do not achieve remission of symptoms after antidepressant treatment [80]. Increasing evidence suggests that epigenetic factors (including DNA methylation and histone modification) play a key role in predicting antidepressant response [80]. The role of small noncoding RNAs (microRNAs, miRNAs) and long noncoding RNAs (lncRNAs) [81] in the aetiology of depressive response is also important. miRNAs can modulate posttranscriptional gene expression by interfering with translation controlled by mRNAs [82]. lncRNAs are highly expressed in the brain and participate in various normal brain functions as well as neuropsychiatric disorders [83]. The role of specific classes of long noncoding RNAs in resiliency or susceptibility to develop depression with a reciprocal response to antidepressant treatment is underlined [83]. Epigenetic changes in the signalling pathway of glucocorticosteroids (e.g., NR3C1, FKBP5), with regard to serotonergic neurotransmission (e.g., SLC6A4) and in the area of genes encoding neurotrophic factors (e.g., BDNF), seem to be the most promising therapeutic targets for future research [23, 84].

## Figures and Tables

**Table 1:** A depressed parent and his/her child [41].

A depressed parent loses interest in the child and its needs. Verbal and nonverbal communication is reduced. Interactions, even if present, are full of tension, sadness, and anger. A depressed parent directs towards the child messages about the lack of faith in the child's abilities and skills. The behaviour of a depressed parent is often inconsistent (from being excessive demanding to excessively lenient). The child observing the parents, through imitation and modelling, takes over stereotypes of thought, models of behaviour and ways of functioning, which become permanent traits of its personality.
